# A case series of blastic plasmacytoid dendritic cell neoplasia

**DOI:** 10.37825/2239-9747.1012

**Published:** 2020-10-01

**Authors:** B Serio, V Giudice, M D’Addona, R Guariglia, M Gorrese, A Bertolini, F D’Alto, B Cuffa, D Pellegrino, M Langella, C Selleri

**Affiliations:** 1 Hematology and Transplant Center, University Hospital “San Giovanni di Dio e Ruggi D’Aragona”, Italy; 2 Clinical Pharmacology, University Hospital “San Giovanni di Dio e Ruggi D’Aragona”, Italy; 3 Department of Medicine, Surgery and Dentistry “Scuola Medica Salernitana”, University of Salerno, Italy

**Keywords:** blastic plasmacytoid dendritic cell neoplasm, flow cytometry, chemotherapy, transplant

## Abstract

Blastic plasmacytoid dendritic cell neoplasm (BPDCN), an extremely rare and aggressive tumor, derives from plasmacytoid dendritic cell precursors and is characterized by CD4 and CD56 positivity accompanied by the expression of isolated myeloid, B- or T-cell lineage markers. Despite the recent introduction of specific targeted therapies, prognosis is still poor with a median overall survival of one year, and allogeneic bone marrow transplantation remains the only curative treatment in eligible patients. In this series, we described two cases of adult BPDCN treated with high dose cytarabine and methotrexate and autologous hematopoietic stem cell transplantation, or fludarabine, cytarabine, and idarubicin achieving the first a complete lasting remission, while the second only a transient improvement in skin lesions.

## I. INTRODUCTION

Blastic plasmacytoid dendritic cell neoplasm (BPDCN), a rare and aggressive hematological disease, has been recognized as a distinct clinical entity in 2016 revision of the World Health Organization (WHO) classification of myeloid neoplasms and acute leukemia [1]. BPDCN mostly affects older males (mean age, 70 years) and is characterized by skin lesions with or without bone marrow (BM) involvement and leukemic cell dissemination in secondary lymphoid organs [1–6]. In addition, 10–20% of patients have a clinical history of other hematological malignancies, such as myelodysplastic syndrome (MDS) or chronic myeloid leukemia [1]. BPDCN cells are of plasmacytoid dendritic cell origins and typically express CD4, CD123 (interleukin-3 a receptor), HLA-DR, cTCL1 (cytoplasmic T-cell leukemia/lymphoma 1) at high levels, CD56, CD304, CD303, CD36, CD38, and CD45RA [7–8]. Chromosomal abnormalities, such as complex karyotype with deletions on chromosomes 5q21 or 5q34, and somatic mutations in genes like *CDKN1B/2A*, *TET2* or *TP53* are also frequently reported [7–11]. To date, there are no guidelines for treatment of advanced BPDCN; however, conventional chemotherapy used for myeloid or lymphoid malignancies are employed with various efficacy [12–13]. Recently, a cytotoxin targeting CD123-expressing cells, tagraxofusp-erzs, has been approved for BPDCN with an overall response rate [ORR] of 90% as frontline therapy or 67% in relapsed/refractory patients [14–15]. However, available studies show that overall survival (OS) is similar between patients receiving conventional chemotherapy and those treated with tagraxofusp-erzs [4]. High dose cytarabine and methotrexate (CVAD) and hematopoietic stem cell transplantation (HSCT) remain the most effective therapies for BPDCN treatment in eligible subjects [4]. With the advances in diagnosis and the appliance of novel targeted regimen, the outcomes for patients with BPDCN may be improved in the future.

In this series, we presented two cases of BPDCN treated at the Hematology and Transplant Center, University Hospital “San Giovanni di Dio e Ruggi d’Aragona” of Salerno after informed consent obtained in accordance with the Declaration of Helsinki [16]. The authors retrospectively reviewed all available medical records.

## II. CASE 1 PRESENTATION

Skin biopsy and immunophenotyping were performed in a 48-year-old female presented with progressive erythematous lesions of lower limbs not improving after a two-year treatment with steroids and colchicine, and a medical history of vitiligo and pityriasis versicolor. She received a diagnosis of BPDCN in April 2014, and leukemic cells were positive for CD4, CD2, CD38, CD56, CD123, and Ki67. No extracutaneous or BM involvements were found after PET-CT scanning and marrow biopsy. She started chemotherapy according to the hyper-CVAD scheme: cycle A with cyclophosphamide, doxorubicin, and vincristine; and four cycles B with methotrexate and subcutaneous cytarabine (Ara-C). Skin lesions gradually disappeared right after chemotherapy, and she was candidate to autologous HSCT. Peripheral blood stem cells (PBSCs) were collected at the end of the third cycle B, and conditioning regimen with busulfan 2 mg/m^2^ and melphalan 130 mg/m^2^ was started after seven months from diagnosis. Post-transplant complications were persistent diarrhea, herpes zoster reactivation, severe peripheral neuropathy, and recurrent bronchitis, all successfully treated. At the time of writing, after six years from HSCT, the patient is alive without signs and symptoms of BPDCN.

## III. CASE 2 PRESENTATION

**Fig. 1:**
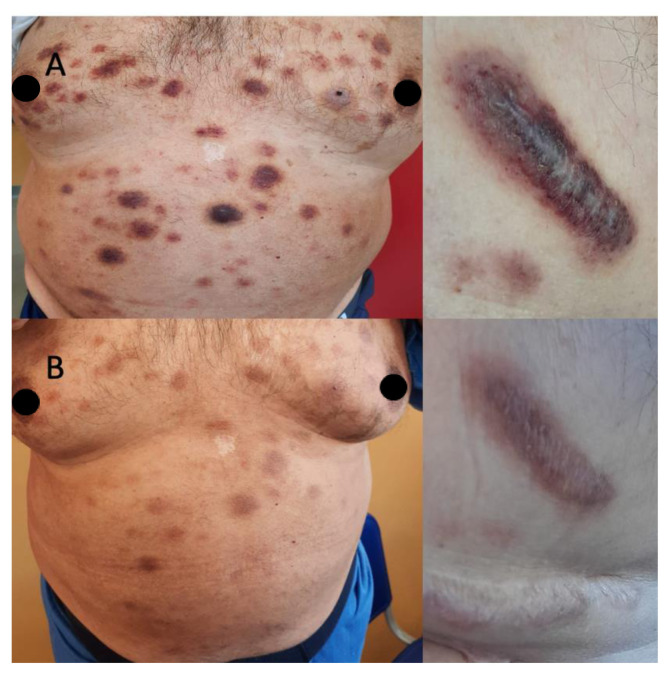
Clinical presentation of Case 2. (A) The patient presented with erythematous infiltrated skin lesions all over the body that were disappearing (B) just after ten days of treatment.

A 55-year-old man received a diagnosis of refractory anemia based on 2016 World Health Organization criteria with an International Prognostic Scoring System (IPSS) of 0 in January 2017 [1]. Peripheral blood and BM flow cytometry immunophenotype showed the presence of aberrant CD14+CD33+CD56+ monocytes. The patient was treated with supportive therapy (epoetin alfa 40.000 IU/weekly) until April 2020 when rapid progressive erythematous skin lesions appeared. Skin biopsy highlighted an infiltration of CD56+CD4+CD3+/− pathological cells with focal expression of MUM1 posing for a diagnosis of BPDCN. No extracutaneous involvements were found after PET-CT scanning, while BM biopsy confirmed the diagnosis of MDS with trisomy 8. Because of low Eastern Cooperative Oncology Group Performance Status (ECOG PS = 1), the patient started a reduced intensity FLAG-IDA protocol (fludarabine, Ara-C, and idarubicin), and skin lesions immediately reduced in number and size (Figure 1). However, blood counts and clinical conditions rapidly worsened after one month, and he died because of disease progression with neurological and hepatic involvement and severe pancytopenia.

## IV. DISCUSSION

BPDCN is a rare disease that behaves like high-risk acute leukemia with poor prognosis because no specific therapies or consensus on frontline treatment are present, and because BPDCN frequently shows chemoresistance. In young patients, intensive induction regimens followed by consolidation and HSCT are considered the most effective treatment strategy, leading to lasting responses, while treatment of elderly patients is still challenging.

In our experience, we presented two adult BPDCN cases treated with hyper-CVAD and autologous HSCT or a FLAG-IDA protocol. The first patient who quickly received HSCT achieved a long-lasting complete remission; however, she had several transplant-related complications. The second case was treated with a reduced intensity FLAG-IDA because of low non-optimal performance status and progression of previously diagnosed MDS. This patient experienced a rapid but transient improvement in skin lesion; however, he died after one month because of disease progression. Therefore, our results confirmed that hyper-CVAD treatment followed by HSCT could be the most effective therapeutic strategy for long-lasting remission in BPDCN patients as previously reported [4]. In particular, allogeneic HSCT recipients have a 10-year OS of 40% with better outcomes when transplant is performed at the first remission [17–18]. In non-eligible patients, autologous HSCT can be an effective alternative, as shown in our case report. Tagraxofusp, bortezomib, azacytidine or venetoclax could also be a valid therapeutic option as preliminary results show significant improvements in ORR [14,19–24]. However, further studies are required to define the best strategy for treatment of BPDCN and to identify candidate biomarkers of responsiveness to therapies.
